# Layer-shaped alginate hydrogels enhance the biological performance of human adipose-derived stem cells

**DOI:** 10.1186/1472-6750-12-35

**Published:** 2012-06-29

**Authors:** Bianca Galateanu, Doina Dimonie, Eugeniu Vasile, Sorin Nae, Anisoara Cimpean, Marieta Costache

**Affiliations:** 1 Department of Biochemistry and Molecular Biology, University of Bucharest, 91-95 Splaiul Independentei, sect 5, Bucharest, Romania; 2 Research and Development National Institute for Chemistry and Petrochemistry, 202 Splaiul Independentei, sect 6, Bucharest, Romania; 3 METAV CD, 31 C. A. Rosetti Street, Bucharest, Romania; 4 Emergency Hospital of Plastic Surgery and Burns, 218 Calea Grivitei Street, sect 1, Bucharest, Romania

**Keywords:** Adipose tissue engineering, hADSCs, Alginate hydrogel, 3-D culture, Adipogenesis, Viability

## Abstract

**Background:**

The reconstruction of adipose tissue defects is often challenged by the complications that may occur following plastic and reconstructive surgery, including donor-site morbidity, implant migration and foreign body reaction. To overcome these problems, adipose tissue engineering (ATE) using stem cell-based regeneration strategies has been widely explored in the last years. Mounting evidence has shown that adipose-derived stem cells (ADSCs) represent a promising cell source for ATE. In the context of a small number of reports concerning adipose tissue regeneration using three-dimensional (3-D) systems, the present study was designed to evaluate the biological performance of a novel alginate matrix that incorporates human ADSCs (hADSCs).

**Results:**

Culture-expanded cells isolated from the stromal vascular fraction (SVF), corresponding to the third passage which showed the expression of mesenchymal stem cell (MSC) markers, were used in the 3-D culture systems. The latter represented a calcium alginate hydrogel, obtained by the diffusion of calcium gluconate (CGH matrix), and shaped as discoid-thin layer. For comparative purposes, a similar hADSC-laden alginate hydrogel cross-linked with calcium chloride was considered as reference hydrogel (RH matrix). Both hydrogels showed a porous structure under scanning electron microscopy (SEM) and the hADSCs embedded displayed normal spherical morphologies, some of them showing signs of mitosis. More than 85% of the entrapped cells survived throughout the incubation period of 7 days. The percentage of viable cells was significantly higher within CGH matrix at 2 days post-seeding, and approximately similar within both hydrogels after 7 days of culture. Moreover, both alginate-based hydrogels stimulated cell proliferation. The number of hADSC within hydrogels has increased during the incubation period of 7 days and was higher in the case of CGH matrix. Cells grown under adipogenic conditions for 21 days showed that both analyzed 3-D culture systems support adipogenic differentiation in terms of neutral lipid accumulation and perillipin expression. Furthermore, the cells encapsulated in CGH matrix displayed a more differentiated phenotype.

**Conclusions:**

The results of this study suggest that both CGH and RH matrices successfully support the survival and adipogenesis of hADSC. An enhancement of biological performance was detected in the case of CGH matrix, suggesting its promising application in ATE.

## Background

In the last decade, advances in bioengineering and cell biology of the adipose tissue have been made and new strategies, which effectively reconstruct the soft tissue defects, have been developed [1,2]. Standard approaches on soft-tissue reconstruction are represented by autologous fat transplantation, alloplastic implants and autologous tissue flaps. However, these approaches have several disadvantages, including donor-site morbidity, implant migration and foreign body reaction. To overcome the limitations of the current restorative techniques, the engineering of the adipose tissue has been proposed as an alternative approach [3,4]. Thus, new tissue-engineered systems for the generation of *de novo* adipose tissue [5,6] are increasingly developing, using the patient’s own fat stem cells.

Adipose tissue-derived stem cells (ADSCs) share many similar characteristics to their counterparts in the bone marrow, including the extensive proliferative potential and the capacity to differentiate into a variety of cell types (adipocytes, osteocytes, chondrocytes, myocytes, and neurons), when cultured with the appropriate stimuli [7-11]. As a result of these features, ADSCs can be used for the regeneration and repair of acute and chronically damaged tissues [12]. In addition to the restorative medicine, ADSCs can be used for cosmetic treatments. Currently, there are two possible tissue engineering strategies to induce *de novo* adipogenesis [13]. One strategy consists in the *in vivo* induction of adipose tissue from precursor or stem cells originally existing in the body. These cells are able to proliferate and mature into adipocytes by creating a biomimetic environment through site-specific delivery of potent bioactive factors [14,15]. The second strategy is to grow *in vitro* the cells isolated from a patient’s own tissue and seed them onto a biocompatible scaffold [12,16,17].

To create adipose tissue-engineered constructs, a diversity of biodegradable natural or synthetic polymer scaffolds has been tested in combination with animal or human adipocyte precursor cells. For instance, such synthetic scaffolds include polylactic-co-glycolic acid (PLGA) [18,19], polyglycolic acid (PGA) [20], and polyethylene terephthalate (PET) [21]. At the same time, a number of biomaterials of natural origin have been investigated for adipose tissue engineering applications, such as collagen sponges [12,22], hyaluronic acid-based scaffolds [23,24], matrigel [25], fibrin [26], and alginate gels [27,28].

The alginate gels cross-linked with calcium ions (Ca2+) have been widely used for tissue engineering studies [29,30] due to their high biocompatibility, relatively low cost, reduced immunogenicity, and capacity of forming hydrogels under gentle conditions. Hydrogel-based materials have been frequently used in tissue engineering applications (especially for soft tissues) due to their particular viscoelastic properties, amiability of fabrication into specific shapes, and ability to form biocompatible solid constructs with homogeneous distribution of cells [31]. Their structures provide encapsulated cells with a 3-D environment similar to that of the extracellular matrix (ECM) of soft tissues, allowing a good transfer of gases and nutrients to maintain cell viability [32], adherence, proliferation and differentiation [33]. Furthermore, the alginate may be easily separated from the embedded cells. Thus, exposed to mild chelating agents, alginate can release the entrapped cells, and exposed to a number of ions, including sodium, the alginate may degrade itself [34].

In a previous report it was shown that attachment-dependent cells are unable to specifically interact with alginate, which promotes minimal protein adsorption, probably due to its high hydrophilic nature [32]. This shortcoming was overcome by modifying substrate surface with a peptide containing the Arg-Gly-Asp (RGD) recognition sequence, known for its ability to mimic extracellular matrix molecule binding sites and stimulate cell adhesion to material [35,36].

Numerous studies regarding cellular behavior in or on unmodified alginate hydrogels have been issued. A considerable number of these studies have been devoted to studying the effects of encapsulation of adult MSCs in alginate gels, especially on chondrogenic [37-39] and osteoblastic differentiation [40].

In the context of a small number of reports concerning adipose tissue regeneration using alginate three-dimensional (3-D) systems, the present study was designed to evaluate the biological performance of a novel alginate matrix that incorporates human adipose-derived stem cells (hADSCs). Therefore, the biological performances of two alginate hydrogel matrices, as temporary physical support for hADSCs, were compared in order to identify an appropriate environment for cell proliferation and adipogenic differentiation. These hydrogels were designed as thin layer disks and prepared by the diffusion of two different cross-linking agents (calcium chloride and calcium gluconate) in cell-loaded alginate solution. The behavior of hADSC cultured under 3-D conditions within alginate hydrogels was analyzed in terms of viability, proliferation, morphology, and adipogenic differentiation.

We found that both calcium gluconate and calcium chloride alginate hydrogels successfully support survival and adipogenic differentiation of hADSC. Moreover, an enhancement of biological performance was detected in the case of CGH matrix, suggesting its promising application in soft tissue engineering.

## Methods

### Primary cultures

The human subcutaneous abdominal white adipose tissue was obtained from moderately overweight women undergoing elective liposuction. All the medical procedures were performed in compliance with the Helsinki Declaration, with the approval of the Emergency Hospital for Plastic Surgery and Burns Ethical Committee (reference no. 3076/10.06.2010). All subjects were in good health and provided their written consent before participating to in the study. None of them had diabetes, severe systemic illness, or was taking medications known as impairing the adipose tissue metabolism. Upon sampling, the lipoaspirates (LAs) were immediately processed for obtaining the stromal vascular fraction (SVF).

hADSCs were isolated as described by Gimble et al. [41]. Briefly, LAs were subjected to collagenase digestion and the obtained SVF was centrifuged at 420 g for 10 min. Then, the pellet was resuspended in growth culture medium (GCM) consisting in Dulbecco’s modified Eagle’s medium (DMEM, Sigma-Aldrich, Co): Ham-F12 supplemented with 1% ABAM, 1.2 g/L NaHCO3 (Sigma-Aldrich, Co) and 0.5 mM sodium pyruvate (Sigma-Aldrich, Co) and seeded at an initial density of 1.5 × 10^4^ cells/cm^2^ in standard cell culture conditions_._ For the first 24 h, the cells have been maintained in GCM supplemented with 40% FBS to allow their attachment and then, this medium was changed to GCM containing 10% FBS.

### Subcultivation

When the cells reached 80% confluence, they were harvested by trypsinization with 0.25% trypsin - 0.5 mM EDTA solution (Sigma-Aldrich, Co). After centrifugation, the cellular pellet was suspended in GCM and plated on T75 cell culture flasks (Nunc) at a cell density of 1.5 × 10^4^ cells/cm^2^. Due to a decrease in the cell proliferation, the culture was propagated up to eight passages. Cell morphology was analyzed every day by phase contrast microscopy (Nikon Eclipse TS 100).

### Characterization of hADSCs cells grown in 2-D culture system

#### Phenotypic characterization

Immunophenotypic characterization of hADSCs at the third passage was achieved by flow cytometry. We examined the expression of the following cell surface antigens: CD34 (mouse anti-human-PE conjugated monoclonal antibody, code A07776, Beckman Coulter) as a hematopoietic marker and CD44 (mouse anti-human monoclonal antibody, 1:50, code Sc-9960, Santa-Cruz Biotechnology), CD73 (rabbit anti-human polyclonal antibody, 1:50, code Sc-25603, Santa-Cruz Biotechnology), CD90 (mouse anti-human monoclonal antibody, 1:50, code Sc-59396, Santa-Cruz Biotechnology) and CD105 (mouse anti-human-PE conjugated monoclonal antibody, code A07414, Beckman Coulter) as typical protein markers in MSCs.

Flow cytometric analysis was issued on a FC 500 Cytometer (Beckman Coulter). Approximately 18000 events were acquired on flow cytometer and analyzed using CXP 2.2 software. The third passage cells were trypsinized, washed twice with 1% bovine serum albumin (BSA, Sigma-Aldrich, Co) solution, and aliquots of 1.4 × 10^5^ cells were incubated at 4°C for 30 min with fluorescent primary antibodies. Upon removal of the excess antibodies by several washes with PBS (Gibco), the samples were subjected to cytometric analysis. Concomitantly, a negative control was prepared by incubating the cells with phycoerythrin (PE) and fluorescein (FITC) conjugated isotype control antibody solution, under the same conditions (IgG1 mouse – PE, code A07796, Beckman Coulter and IgG1 mouse – FITC, code A07795, Beckman Coulter).

### 3-D cell cultures within alginate hydrogel matrices

Third passage cells were detached from the monolayer by trypsin-EDTA treatment, centrifuged, counted and mixed with sterile 1.5% (w/v) low viscosity sodium alginate in 0.9% NaCl at a concentration of 7 x 10^5^ cells/ml. The cell-alginate mixture was distributed into the wells of a 6-multiwell culture plate (Nunc) for further flow cytometric studies. For microscopy studies and spectrophotometric MTT (3-(4,5-dimethylthiazol-2-yl)-2,5-diphenyltetrazolium bromide) assay, the cell suspension was distributed into the wells of a 12 multi-well culture plate (Nunc). To produce alginate gel, a sterile disc of Whatmann filter paper, was soaked with the cross-linking agent and placed above the alginate-cell solution. Equal volume of gelling agent was placed above the disk, and plates were incubated for 45–60 min at 37°C in a humidified atmosphere of 5% CO_2_. After gelling, the paper disks were removed and the remaining fluid was aspirated. The resulting thin layer alginate hydrogels were sequentially washed with 0.9% saline solution and GCM, covered with culture medium, and subjected to incubation in standard conditions.

Herein, we used two gelling agents: (1) calcium chloride solution (Sigma-Aldrich, Co) currently used for obtaining cell-laden alginate hydrogels and considered as the reference hydrogel material, and (2) calcium gluconate solution (Zentiva), which is mainly used in preparing the alginate matrix of Drug Delivery Systems. The detailed procedure will be the subject of a patent.

### Characterization of hADSCs incorporated in 3-D alginate systems

#### Scanning electron microscopy (SEM)

The hADSCs-laden alginate hydrogels, maintained in culture for 2 and 7 days, were washed twice with PBS and fixed for 6 h at 4°C with 2.5% glutaraldehyde (Sigma-Aldrich, Co) in PBS, containing 0.1% CaCl_2_. After rinsing with double distilled water, the samples were dried at 20°C and 0.1 mbar pressure for 4 hours in a 24-LSC Martin Christ laboratory freeze dryer. Then, the samples were coated with gold and imaged using a FEI Quanta Inspect F with field emission gun (FEG), operating in Scanning Electron Microscopy (SEM) mode. The microscope was driven with an acceleration voltage of 30 kV and a working distance of 10 mm detecting secondary electrons.

#### Assessment of cell viability and proliferative activity

Cell viability within the alginate-based 3-D culture systems was evaluated by flow cytometric determination of live versus dead cells recovered from the hydrogel and stained using Live&Dead Kit (Invitrogen). This method allows the simultaneous detection of both live and dead cells with calcein acetoxymethyl calcein AM and ethidium bromide dyes provided in the kit. Calcein AM is a non-fluorescent and permeable reagent, which is converted by the intracellular esterases to the intensely green fluorescent calcein (ex/em: ~ 495 nm/~ 635 nm). Ethidium bromide enters the cells with damaged membrane, producing a bright red fluorescence when binding to nucleic acids (ex/em: ~ 495 nm/~ 635 nm).

Briefly, at 2 and 7 days post-seeding, the two alginate-based hydrogels were first solubilized by incubation for 15 min at 37°C in 94 mM NaCl solution containing 350 mM sodium citrate (Sigma-Aldrich, Co) and 35 mM MOPS (Sigma-Aldrich, Co). After 10 min centrifugation at 265 g, 1.4 x 10^5^ cells were incubated at room temperature in the dark with calcein AM and ethidium bromide solutions contained in the kit. A mean value of 10.000 events was acquired by FC500 flow cytometer (Beckman Coulter) and analyzed with CXP 2.2 software (Beckman Coulter).

The proliferation capacity of the cells within the alginate hydrogels was assessed by MTT assay at 2 and 7 days post-seeding. Briefly, both RH and CGH matrices were incubated for 15 h in MTT solution (1 mg/ml in serum free culture medium) in standard conditions of cultivation. Before dissolving the formazan crystals produced by the metabolically active cells with isopropanol, the images of MTT-stained culture were taken using an Olympus IX71 inverted microscope. Solubilization was followed by spectrophotometric quantification at 550 nm.

#### Assessment of adipogenic differentiation

The third passages ADSCs embedded in either RH or CGH matrices were analyzed for their capacity to differentiate towards the adipogenic lineage. The adipogenic differentiation was induced by culturing subconfluent cells in a new formula of adipogenic differentiation medium (ADM), consisting in GCM supplemented with an adipogenic cocktail. This experiment was conducted over a 21-day incubation period. The adipogenic differentiation was quantified by the cellular capacity to accumulate intracellular lipids and by perilipin expression level. A detailed description of the ADM medium and of the entire experimental procedure will be provided in a patent application.

The accumulation of cytoplasmic droplets of neutral lipids was assessed by Oil Red O staining. The alginate-embedded cells cultured in 12-well plates were allowed to accommodate for 3–4 days in the new 3-D microenvironment. Subsequently, the GCM was supplemented with the adipogenic cocktail.

The cytoplasmic lipid droplets visualization was issued at different time points, as described by Brandl et al. [15], with modifications. Briefly, the cell-laden alginate hydrogels were washed with PBS and fixed for 8 h with 4% PFA. After permeabilization with 2% BSA/0.1% Triton X-100 solution, both CGH and RH matrices were incubated with Oil Red O (5 mg/ml in 60% isopropanol, diluted 3:2 with tap water) for 24 h at 4°C. Upon removal of the Oil Red O solution, the hydrogels were washed several times to eliminate the traces of staining solution. The assessment of lipid droplets accumulation was revealed by phase contrast microscopy (Olympus IX71) and Cell F Imaging Software (Olympus).

For flow cytometric detection of perilipin at 3, 7, 15 and 21 days after adipogenic induction, 3-D culture systems were first solubilized as previous described. After 10 min centrifugation at 265 g, 1.4 x 10^5^ cells were fixed with 4% PFA and permeabilized with 2% BSA/0.1% Triton X-100 solution. After a short washing step, the cells were incubated overnight with rabbit polyclonal anti-perilipin antibody solution (1:200, Santa-Cruz Biotechnology) and for 30 min with FITC conjugated goat anti-rabbit IgG1 secondary antibody (1:50, Santa-Cruz Biotechnology). A mean value of 10000 events was acquired and analyzed with CXP 2.2 software (Beckman Coulter). In order to keep the same parameters throughout the entire culture period, the cytometer was calibrated with Flow Check fluorescent beads (Beckman Coulter) before each determination.

## Results

### Primary culture and characterization of hADSC in a 2-D culture system

hADSCs cultures were isolated and purified from human subcutaneous adipose tissue, which was obtained by elective liposuction. The primary culture was obtained by collagenase digestion of the adipose tissue, followed by the seeding of the heterogeneous cell suspension, and subsequent removal of non-adherent cells. As the cells were propagated in monolayer culture, they showed a more uniform fibroblast-like morphology, suggesting the existence of a homogenous ADSC cell population.

#### hADSCs cell surface antigen expression

Given the cell heterogeneity of LAs and of primary culture, serial passages were analyzed for the expression of hADSC-associated markers. The third passage cells were determined as being the most suitable for the incorporation into the alginate matrix (data not shown).

**Figure 1:**
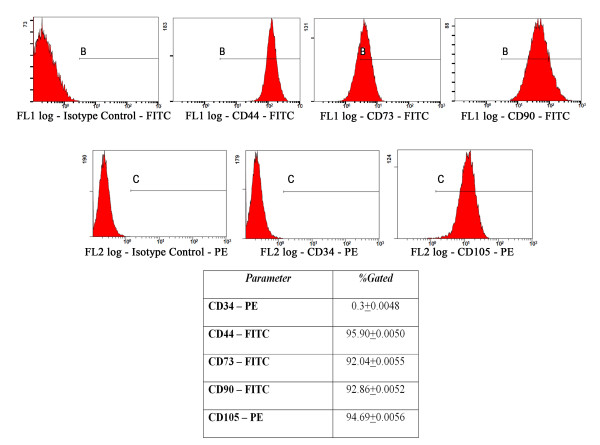
**Immunophenotypic analysis of the third passage hADSCs by flow cytometry.** The fluorescence level detected for the stromal stem cells markers was 95.9% for CD44, 92.04% for CD73, 92.86% for CD90 and 94.6% for CD105, respectively, whereas for the hematopoietic stem cells marker CD34 only 0.3% of the gated cells were fluorescently labeled. The positive zones in the histograms were marked with B in the fluorescence channel 1 (FL1), and with C in the fluorescence channel 2 (FL2), respectively, based on the isotype control antibody.

The flow cytometric data revealed that the third passage hADSCs are negative for the hematopoietic marker CD34 and positive for the stromal cell markers CD44, CD73, CD90 and CD105, which are common to human bone marrow-multipotent MSCs (hBM-MSCs) (Figure 1). Thus, more than 90% of these cells purified by conventional culture methods expressed CD44, CD73, CD90 and CD105 respectively, while only 0.26% of the cells expressed CD34, which is selectively expressed on hematopoietic progenitor cells and vascular endothelium.

These data suggest that the third passage hADSCs exhibit a MSC origin, and that they are not intermingling with hematopoietic cells. Based on these results, we established that the most suitable hADSCs for incorporation into the alginate matrix were the cells corresponding to the third cell passage.

### *In vitro* behavior of hADSC embedded into alginate hydrogels

#### Macroscopic and microscopic evaluation of the cell-laden alginate hydrogels

**Figure 2:**
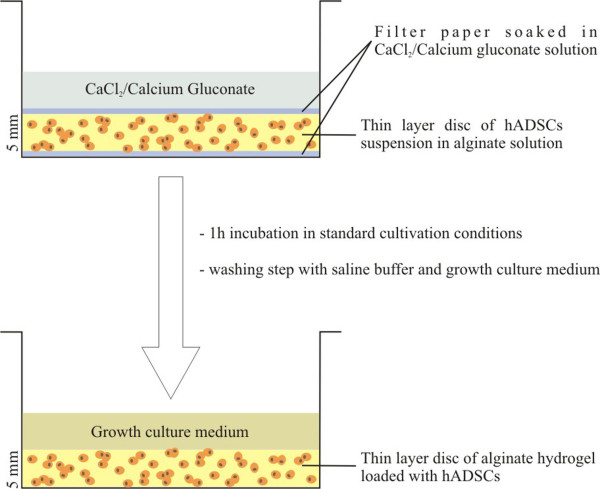
**Schematic diagram showing the preparation of the cell-laden layer-shaped alginate hydrogels.** STEP 1: A filter paper soaked in the cross-linking agent (CaCl_2_ or calcium gluconate) was placed on the bottom of each well of a 6-well culture plate. The alginate-hADSCs suspension was added above and covered with the second filter paper soaked in the same gelling agent. CaCl_2_ or calcium gluconate was added on the top of the construct and the plate was incubated for 45–60 min at 37°C in a humidified atmosphere of 5% CO_2_; STEP 2: The excess of the cross-linking agent was removed together with both of the filter papers used. The resulting thin layer hydrogel (5 mm thickness) was washed extensively with saline buffer and GCM.

The hADSC-laden hydrogels were poured into 12 and 6-well plates and were constructed as disks of 5 mm thickness. Throughout the entire culture period these matrices have been stable, have not exhibited any contraction, and could be easily handled. A schematic diagram of the 3-D cell culture system preparation is shown in Figure 2.

**Figure 3:**
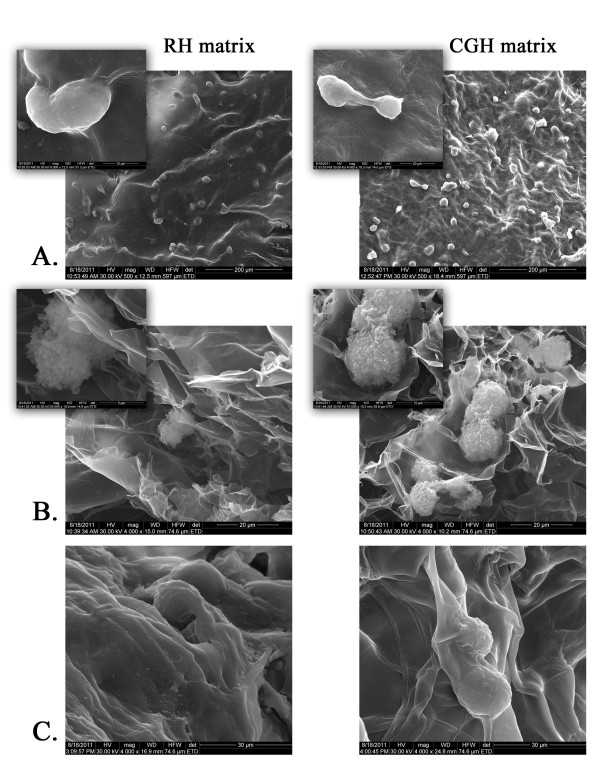
**Scanning Electron Microscopy (SEM) micrographs of both 3-D porous cell-laden RH and CGH matrices.** A) Surface appearance of RH and CGH matrices after 2 days of culture; B) Cross-sections of the cell-laden 3-D structures at 2 days post-seeding; C) Cross-sections of cell-laden hydrogels at 7 days post-seeding.

The morphology of the cell-laden alginate hydrogels was examined using SEM after 2 and 7 days of culture, respectively. The investigation of the construct surface after 2 days of cultivation revealed the presence of numerous cells in various stages of mitosis, entrapped within the alginate matrix (Figure 3A). SEM images of relevant cross-sections showed that hADSCs were distributed within the pores of the matrix and exhibited a round-shaped morphology due to the lack of interactions between cells and alginate polymer (Figure 3B). The examination of the cross-sections of cell-laden hydrogels at 7 days post-seeding showed that hADSCs remained viable in these matrices, showed signs of mitosis, and seemed to progressively produce and organize their own extracellular matrix (Figure 3C).

#### hADSCs viability and proliferation in the alginate hydrogels

**Figure 4:**
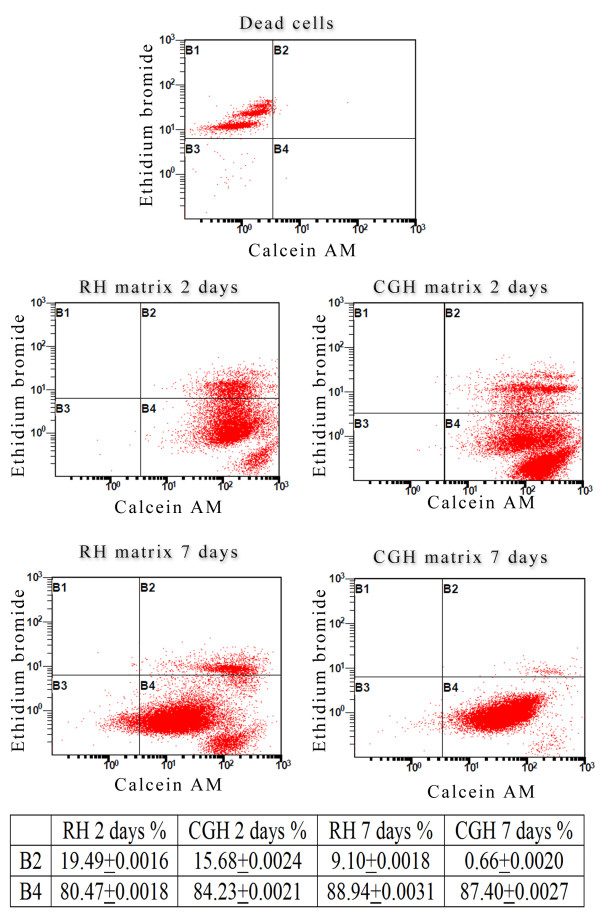
**Flow cytometric evaluation of hADSCs viability in both RH and CGH matrices.** Fluorescence channel 1 (FL1)/Fluorescence channel 2 (FL2) plots showing Ethidium Bromide positive cells (B1), both Ethidium Bromide and Calcein AM positive cells (B2), unmarked debris (B3) and Calcein AM positive cells (B4).

In order to examine cell survival during culture, the viability of the hADSCs embedded in alginate and recovered by hydrogel solubilization was evaluated at both 2 and 7 days post-seeding, using a LIVE/DEAD assay. Flow cytometric analysis of the labeled cells revealed that at both time points, more than 80% of the gated cells were positive for the cell viability marker calcein AM, as shown in Figure 4, B4 region of the dot-plots. Two days post-seeding, 19.49% of hADSCs embedded into RH alginate matrix and 15.68% of the cells embedded into CGH matrix were found positive for both calcein AM and Ethidium bromide, suggesting their plasma membrane alteration. Interestingly, after 7 days of culture, the percentage of double-labeled embedded cells decreased to 9.1% for the RH matrix and only to 0.66% for the CGH matrix, respectively (Figure 4 - B3 region of the dot-plots). This could be due to the accommodation of the entrapped cells to the new 3-D culture conditions***.***

**Figure 5:**
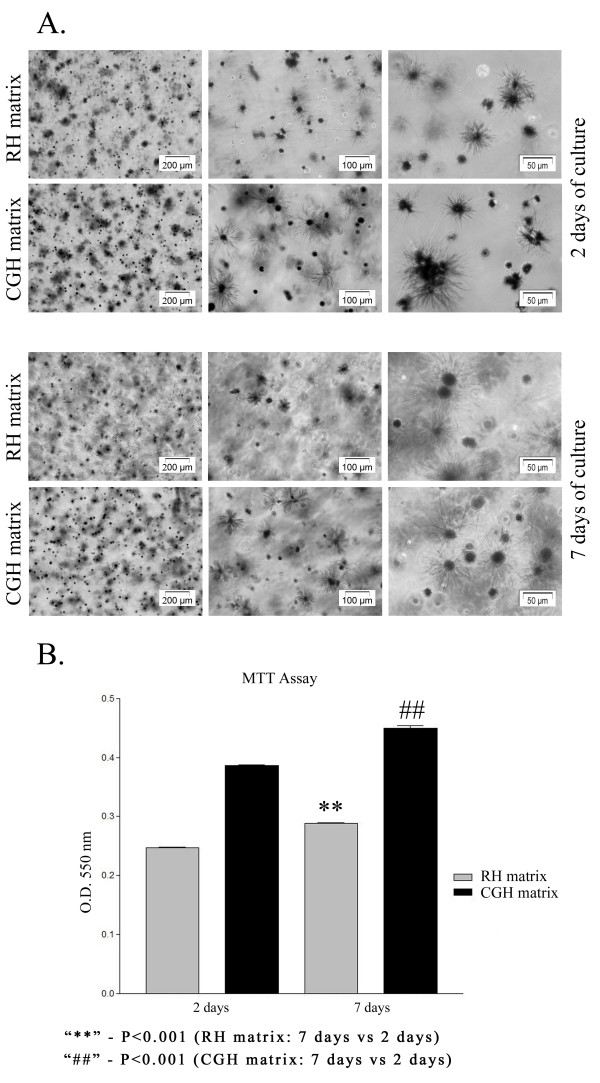
**Proliferation potential of hADSCs within RH and CGH matrices.** A) Phase contrast micrographs of MTT-stained viable hADSCs embedded in either RH or CGH matrices: UP: Phase contrast micrographs of MTT-stained viable hADSCs embedded in RH and CGH matrices after 2 days of culture showing a higher number of living cells in CGH matrix compared to RH matrix. DOWN: Phase contrast micrographs of MTT-stained viable hADSCs embedded in RH and CGH matrices after 7 days of culture showing an increasing number of living cells in both constructs as a result of cellular proliferation. B) hADSCs proliferation within RH and CGH matrices, measured by formazan absorbance: 2 and 7-days post-seeding. The proliferation rate in CGH matrix at 2 days of culture is 43.3% higher as compared to the RH matrix. At 7 days post-seeding CGH matrix contains 44.6% more viable cells than RH matrix. Both culture systems ensured a proliferation of approximately 17% at 7 days post-seeding as compared to a culture of 2 days. Results are presented as means ± SD (n = 3).

To validate the viability/proliferation rate, MTT assay was employed as a more accurate approach. This test is based on the reduction of a tetrazolium salt solution - MTT to purple formazan by metabolic active cells. The precipitated formazan is then solubilized, and the concentration determined by optical density at 550 nm. The result is a sensitive assay with a colorimetric signal proportional to the cell number. Phase contrast micrographs of cell-laden hydrogels after MTT staining revealed the presence of metabolically active cells, at 2 and 7 days of culture, respectively (Figure 5A). A higher number of viable cells converting MTT to formazan crystals were noticed in the CGH matrix as compared to the RH matrix. This finding is in agreement with the results obtained from the LIVE/DEAD assay.

In addition, a spectrophotometric determination of formazan concentration was issued at the same intervals of time. As shown in Figure 5B, at 2 days of culture, the number of viable metabolically active cells within CGH matrix was higher by 43.3% (p < 0.001) than in RH matrix, while after 7 days of culture this increase was 44.6% (p < 0.001). Moreover, hADSCs demonstrated their ability to proliferate in both analyzed matrices. Indeed, the formazan absorbance measured at OD_550_ at 7 days post-seeding was approximately 17% (p < 0.001) higher as compared to the values registered at 2 days post-seeding.

### Adipogenic differentiation of hADSCs in the 3-D culture systems

In order to prove the potential of hADSCs to undergo adipogenic differentiation within alginate hydrogels, GCM was exchanged for the ADM after 3 days of incubation. Intracellular lipid droplet accumulation and perilipin expression have been evaluated at different intervals of time during the incubation period of 21 days for both RH and CGH matrices.

**Figure 6:**
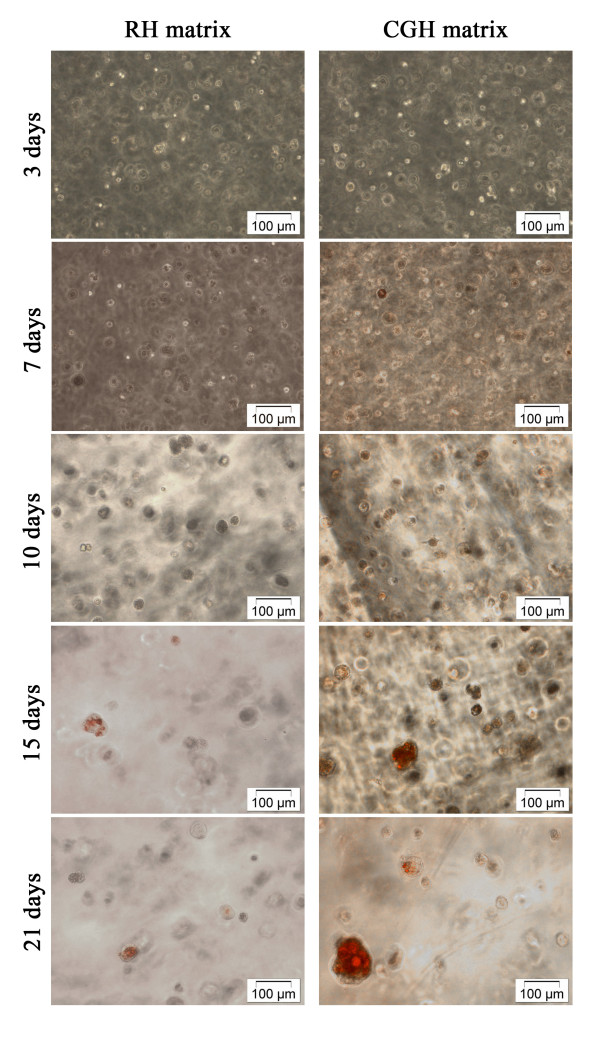
**Phase contrast images of hADSCs embedded in RH and CGH and subjected to adipogenesis.** Both cell-laden alginate constructs were stained with Oil Red O after 3, 7, 10, 15 and 21 days of adipogenesis, to visualize the size and the amount of cytoplasmic lipid droplets

The results of Oil Red staining showed that in the cells embedded in CGH matrix the accumulation of lipids begins at 7 days after adipogenic induction, while for the cells embedded in the RH matrix, a positive staining was observed at 15 days of adipogenic treatment (Figure 6). Furthermore, hADSCs embedded in CGH matrix were more intensively stained and presented clusters of lipid droplets larger than those seen in the RH matrix. Based on these data it can be concluded that the cells encapsulated in CGH matrix produced more lipid droplets and had a more differentiated phenotype.

In order to prove whether RH and CGH matrices are effective for the adipogenic differentiation, the level of perilipin expression in hADSCs recovered after hydrogel solubilization was detected by flow cytometry.

**Figure 7:**
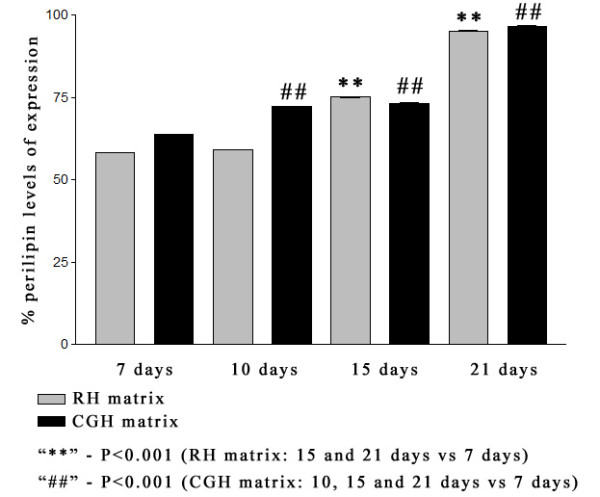
**Flow cytometric results showing the expression of perilipin in hADSCs embedded in alginate hydrogels during adipogenesis.** The fluorescence level detected for perilipin in fluorescence channel 1 (FL1) was increasing in time for both 3D culture systems. Highly significant differences in perilipin expression levels were found at 21 days of induction compared to one week of adipogenesis, in both RH and CGH matrices. In RH matrix, lower values of fluorescence levels were recorded for 7 and 10 days of adipogenic induction as compared to CGH matrix. No significant differences between RH and CGH matrices were reported in perilipin expression after 2 weeks of adipogenesis. Results are presented as means ± SD (n = 3).

The expression levels of perilipin have increased significantly during the culture period, reaching a maximum value of 95.12% for the RH matrix and 96.59% for the CGH matrix, respectively, after 21 days of adipogenic induction (Figure 7).

The fluorescence level detected for perilipin expression was increasing in time for both 3-D culture systems. In RH matrix, lower values of fluorescence levels were recorded for 7 and 10 days of adipogenic induction as compared to CGH matrix: 58.27% and 59.1% compared to 63.81% (p < 0.001) and 72.14% (p < 0.001) respectively. Constant values of perilipin expression were recorded between CGH and RH matrices after 2 weeks of adipogenesis: 75.12% in RH matrix compared to 73.22% in CGH matrix after 15 days and 95.12% in RH compared to 96.59% in CGH after 21 days of induction.

The results of MTT spectrophotometric assay and flow cytometric data of perilipin expression were statistically analyzed with GraphPrism 3.03. Software using One-way ANOVA with Bonferroni’s multiple comparison tests. All results are expressed as means ± SD (standard deviation) and differences at p ≤ 0.05 were considered statistically significant.

## Discussion

Despite intensive work on stem cell-based adipose tissue engineering [1,2,42] much remains to be unraveled regarding the role of the scaffold and culture environment required for the commitment of the undifferentiated cells to the adipogenic differentiation program. Herein, we sought to test a novel cell-laden hydrogel construct that incorporates hADSCs in a calcium alginate gel network. This matrix was shaped as discoid-thin layer obtained by diffusion of calcium gluconate (CGH) and calcium chloride solution (reference hydrogel - RH), respectively, through a filter paper placed above the cell loaded alginate solution. We have chosen the alginate as a cell carrier material since it is a natural polysaccharide abundantly available in nature, which forms hydrogels under relatively mild pH and temperature conditions. In addition, the alginate is suitable for sterilization and storage for long periods of time prior to its use for cell encapsulation [43]. Furthermore, it is a biodegradable and non-toxic material [32], as the water-soluble alginate chains are excreted by the kidney [44].

Cross-linking of alginate with CaCl_2_ has been mainly employed to prepare hydrogel-based cell carrier systems shaped as spherical beads [45-47], disks [48] and thin membrane layers [49]. Due to the very rapid binding of Ca^2+^ to the G-units, it is impossible to obtain a homogeneous gel by simply adding CaCl_2_ to an alginate solution. Kuo and Ma [50] described a new method of internal gelation by controlling the gelation rate that uses a less soluble calcium salt (CaCO_3_) and d-glucono-δ-lactone (GDL). The latter was partially hydrolyzed to gluconic acid, which slowly released small amounts of Ca^2+^ from CaCO_3_ and allowed the formation of a homogeneous gel. In this study, we developed a procedure to prepare alginate hydrogels that does not involve direct mixing of the alginate solution containing cells with the reticulating agent. We have chosen calcium gluconate as a cross-linking agent because it represents the least toxic form of ionized calcium to cells and tissues and it seems to provide a moderate level of Ca^2+^. Moreover, this compound was successfully used in preparing the alginate matrix of drug delivery vectors [51-54]. Although the impact of calcium gluconate was not clearly elucidated, Gungor et al. [52] reported that drugs can be released both faster and in a higher amount from the alginate matrices containing calcium gluconate at pH 7.4. This process was due to the channeling effect of this soluble salt, which increased gel porosity [55].

To gain more insight into the property of calcium gluconate as a cross-linking agent, we analyzed the *in vitro* behavior of hADSCs into this matrix. The hADSCs were thus embedded into this alginate hydrogel, and their morphology, viability, proliferation capacity and adipogenic differentiation were monitored with respect to a reference hydrogel.

The hADSCs were purified by sequential passage of SVF cells in a culture medium specific for the mesenchymal cell lineage. Thus, a homogenous adherent cell population exhibiting a spindle-shaped fibroblast morphology previously reported for stem cells was noticed starting with the third passage cells [56]. As previously reported, the hADSCs could be readily expanded in culture [3]. They have been shown to exhibit two important properties: (1) proliferation in sufficient amounts to produce cell mass for therapeutic purposes and (2) differentiation into different cell types.

However, the disadvantage of using the ADSCs in tissue engineering is that these cells have a limited capacity for sub-culturing. Based on morphological features and phenotype analysis by flow-cytometry of culture-expanded cells isolated from the SVF, the cell population corresponding to the third passage, showing the expression of MSC markers, was used in the 3-D culture systems.

To date, there are very few reports on the adipose tissue engineering and soft tissue regeneration using alginate systems as cell carrier materials for hADSCs. For instance, a study issued by Jing et al. [28] demonstrated that mouse pre-differentiated ADSCs embedded into an alginate-based construct had the capacity to form ectopic adipose tissue when implanted into nude mice. To further elucidate the properties of the hADSCs in adipose tissue engineering, we studied the morphology of the hADSCs embedded in both RH and CGH matrices. Electron microscope images of the two alginate-based hydrogels showed a porous structure, whereby the hADSCs display normal spherical morphologies, some of them showing signs of mitosis.

In order to examine cell survival during culture, the viability of the encapsulated hADSCs in the alginate hydrogels was evaluated after 2 and 7 days of culture by a flow cytometry-based LIVE/DEAD assay. At both analyzed time-points, most of the entrapped cells (over 80%) successfully survived within RH and CGH matrices. Furthermore, the percentage of viable cells was significantly higher within CGH matrix at 2 days post-seeding and approximately similar within both hydrogels after 7 days of culture. These results are in keeping with the observation that the alginate matrices present a structure formed by interconnected pores, which is suitable to accommodate the hADSCs. Furthermore, these matrices successfully supported their viability, nutrient and protein transport.

To get a more complete image on the cell survival and proliferation of hADSC embedded in RH and CGH matrices, microscopic- and spectrophotometric-MTT based assays were issued at the same time intervals. These analyses provided evidence that both alginate-based hydrogels stimulated cell proliferation, the number of hADSCs within hydrogels increasing with the length of the incubation period. This finding is contrary to other studies showing that alginate hydrogels do not allow or inhibit proliferation and growth of several different types of cells when they are either grown on their surface as 2-D monolayer culture or incorporated into the matrix of the gel [57]. Interestingly, in our study, a higher number of metabolically active cells was found within the CGH matrix rather than the RH matrix. Therefore, the growth and metabolic activity of hADSCs seemed to be influenced by the alginate cross-linking agent. The better cell survival and growth-supporting activities of the CGH matrix could be explained by the larger pore sizes than in the case of RH matrix (data not shown). Due to this particular structure, a more efficient transport of oxygen and nutrients may take place in the hydrogel matrix.

To evaluate the effect of calcium gluconate as alginate reticulating agent on adipogenesis, hADSCs encapsulated in alginate hydrogels were cultured with an adipogenic medium up to 21 days. Typical markers of lipid biosynthesis were analyzed. Thus, results of the Oil Red O staining showed that hADSCs embedded in CGH matrix started the process of neutral lipid accumulation at 7 days post-adipogenic induction, whereas in the cells embedded in RH matrix, a positive Oil Red O staining was observed after 15 days of adipogenic induction. This result demonstrated that in CGH matrix a greater number of cells have undergone adipogenesis within 21 days of induction, as compared to cells embedded in RH matrix. Flow cytometric detection of perilipin expression also confirmed a more rapid induction of adipogenesis in CGH matrix compared with RH matrix. The delay in the adipogenic induction of hADSC embedded in RH matrix could be explained by the complexity of the effects that Ca^2+^ exerts on the adipogenesis process [58-60]. Thus, extracellular Ca^2+^ concentration ([Ca^2+^e) can modulate many aspects of cellular behavior, such as: proliferation, differentiation, survival, and death. Jensen et al. (2004) have shown that the levels of [Ca^2+^e are important in regulating adipocyte lipid accumulation. Furthermore, they have demonstrated that increasing intracellular Ca ions ([Ca^2+^i) in early stages of differentiation suppressed human adipocyte differentiation. Accordingly, the present study demonstrates a faster expression of adipogenic markers (intracellular lipid droplets accumulation and perilipin expression) in CGH matrix compared with the reference hydrogel, probably due to a slower eliberation of the calcium ions. In addition, we cannot rule out a possible influence of [Ca^2+^ on the cell sensitivity to insulin, an adipogenic inductor contained by ADM, as it is largely accepted that increased [Ca^2+^ contributes to insulin resistance [61].

Since perillipin is not expressed before adipogenic differentiation [62], its high expression in the hADSCs embedded in CGH matrix suggests that calcium gluconate is effective for the adipogenic differentiation of hADSCs in this 3-D culture system.

## Conclusions

A hADSC-laden alginate hydrogel shaped as thin layer disk was developed by diffusion of a new compound, calcium gluconate, within the alginate gel matrix. In addition, we investigated whether calcium gluconate had a positive effect as alginate cross-linking agent on cell viability, proliferation and adipogenic differentiation in comparison with a reference matrix. Our results clearly demonstrate that both alginate microenvironments support hADSCs viability and proliferation. These matrices do not alter the cell morphology and create conditions that are favorable for adipogenic differentiation. Furthermore, an enhancement of all these cellular parameters was found out in the case of alginate hydrogel obtained by using calcium gluconate as reticulating agent, suggesting its promising application in soft tissue engineering.

## Abbreviations

ADSC, adipose-derived stem cells; ABAM, antibiotic antimycothic; ADM, adipogenic differentiation medium; BSA, bovine serum albumin; CD, cluster of differentiation; CGH matrix, calcium gluconate hydrogel matrix; FBS, Fetal Bovine Serum; FITC, Fluorescein isothiocyanate; GCM, Growth Culture Medium; GDL, d-glucono-δ-lactone; hADSC, human adipose-derived stem cells; hBM-MSCs, human bone marrow-multipotent mesenchymal stem cells; LAs, Lipoaspirates; MOPS, 3-(N-morpholino)propanesulfonic acid; MSC, Mesenchymal Stem Cells; MTT, 3-(4,5-Dimethylthiazol-2-yl)-2,5- diphenyltetrazolium bromide; O.D., Optic density; PBS, Phosphate Buffer Salt; PE, Phycoeritrine; PET, Polyethylene terephthalate; PGA, polyglycolic acid; PLA, Processed lipoaspirate cells; PLGA, Polylactic-co-glycolic acid; RGD, Arginine-glycine-aspartic acid; RH matrix, Reference Hydrogel matrix; SEM, Scanning Electron Microscopy; SVF, Stromal Vascular Fraction.

## Competing interests

Hereby, we Bianca Galateanu, Anisoara Cimpean and Marieta Costache declare that we are University of Bucharest employees main grant recipient of project PCCE 248/2010 whereby this study was financially supported and whereby this paper fee will be assured in case of acceptance. Therefore we have no competing interests.

Hereby, the undersigned Doina Dimonie I declare that the 3-D system preparation method is protected by a national patent request. Therefore I have no competing interests.

Hereby, Eugeniu Vasile and Sorin Nae declare that we have no competing interests.

## Authors’ contributions

BG carried out the isolation of human adipose-derived stem cells from lipoaspirate and all the procedures performed in the cell culture field. She performed the phase contrast and fluorescence microscopy, the flow cytometry and the spectrophotometric assays and also carried out the statistical analysis. She was involved in the hydrogels design and preparation and helped to draft the manuscript. DD is responsible for the design of the 3-D thin layer alginate hydrogels. EV carried out the SEM assay. SN provided the lipoaspirate in accordance with the EU ethical standards. MC and AC contributed equally to this work. They were responsible for the coordination of the whole study, for the interpretation of the results and for drafting the manuscript. They also assured a good communication between authors. All authors read and approved the final manuscript.

## Authors’ information

**BG** is a PhD Student in the last stage of the internship and researcher in the Department of Biochemistry and Molecular Biology. Her PhD thesis involves the development of new strategies in adipose tissue engineering using precursor cells and natural biocompatible polymers. Her interest in the field of tissue bioengineering was well evaluated at International Congresses.

**DD** is senior researcher at ICECHIM. She has competences in obtaining and studying new eco - friendly polymeric materials for eco - friendly applications. She has a long experience in this field that means papers as main author in ISI coated journals, patents as main author, new products and technologies as project manager.

**EV** is PhD Engineer senior researcher with high specialization in microscopy.

**SN** is a physician in the field of Plastic Surgery and also scientific researcher. He works within the Emergency Hospital for Plastic and Reconstructive Surgery and Burns in Bucharest and he collaborates with numerous aesthetic surgery private clinics.

**AC** is associate professor in the University of Bucharest and Scientific Researcher in the Department of Biochemistry and Molecular Biology. She attended a post-doc fellowship in Leuven, Belgium and leads research projects concerning the field of tissue bioengineering.

**MC** is full professor at the University of Bucharest, Head of the Department of Biochemistry and Molecular Biology and director of numerous national and international grants. One of the most important research projects led by her is the one financing this study. She obtained her PhD degree in Molecular Biology of the Cell in Paris, France, in 1997.
